# Genetic Diversity of *Puumala orthohantavirus* in Rodents and Human Patients in Austria, 2012–2019

**DOI:** 10.3390/v13040640

**Published:** 2021-04-08

**Authors:** Jeremy V. Camp, Eva Schmon, Robert Krause, Wolfdieter Sixl, Daniela Schmid, Stephan W. Aberle

**Affiliations:** 1 Center for Virology, Medical University of Vienna, 1090 Vienna, Austria; Jeremy.Camp@meduniwien.ac.at; 2 Institute of Hospital Hygiene and Microbiology, Styrian Hospital Corporation, 8010 Graz, Austria; Eva.Schmon@kages.at; 3 Section of Infectious Diseases and Tropical Medicine, Department of Internal Medicine, Medical University of Graz, 8036 Graz, Austria; Robert.Krause@medunigraz.at; 4 Institute of Hygiene, University of Graz, 8010 Graz, Austria; Wolfdieter.Sixl@chello.at; 5 Austrian Agency for Health and Food Safety (AGES), 1090 Vienna, Austria; Daniela.Schmid@ages.at

**Keywords:** hantavirus, *Puumala orthohantavirus*, genetic diversity, phylogeography, molecular epidemiology

## Abstract

*Puumala orthohantavirus* (PUUV) has a wide distribution throughout Europe. Distinctive temporal patterns of spillover into the human population are related to population dynamics of the reservoir host, the bank vole (*Clethrionomys glareolus*). As the rodent host is tied to specific habitats with small individual ranges, PUUV genetic diversity is also highly correlated with geographic distance. Using sequenced portions of viral S and M segments, we determined whether geographic clusters were supported. Human cases of PUUV infections are concentrated in southeastern Austria. We detected four distinct genotypes: two genotypes of the Alpe-Adria (ALAD) lineage typically associated with southeast Europe, and two sublineages of the Central Europe (CE) lineage. One cluster of CE genotypes represents a phylogenetically distinct sublineage compared to previously reported CE clades, and extends the boundary of the CE lineage further south than previously reported.

## 1. Introduction

Hantaviruses (family *Hantaviridae*) are a diverse group of enveloped, single-stranded, negative sense RNA viruses in the order *Bunyavirales*. They are primarily rodent-borne viruses, although shrew-, mole- and bat-borne hantaviruses have also been recently discovered [1]. Infection causes no apparent disease in their reservoir hosts, indicating a high level of adaptation to host species. This high degree of adaptation is also reflected in the phylogeography of hantaviruses, where two or more virus species may exist in their respective sympatric rodent species. In general, genetic diversity within a given species of hantavirus is also highly correlated with geography, as rodents often have relatively small ranges [2]. Thus, there exists a highly diverse array of hantavirus species world-wide, each tied to given reservoir host species.

In Europe, there are at least four species of hantavirus—*Dobrava-Belgrade orthohantavirus*, *Puumala orthohantavirus* (PUUV), *Seoul orthohantavirus*, and *Tula orthohantavirus*—circulating in their respective rodent populations, and more species with potential human importance have been recently recognized, particularly among rodents and insectivores [3,4,5]. These viruses may cause mild-to-severe haemorrhagic fever with renal syndrome in humans. Spillover into humans occurs from direct contact with rodents or inhalation of aerosolized excreta (urine or faeces). Therefore, incidence in the human population is related to rodent presence/abundance in domestic or peridomestic locations [1].

Among hantaviruses circulating in Europe, PUUV is the most widespread, owing to the comparatively large geographic distribution of the bank vole, *Clethrionomys glareolus* (Schreber, 1780) (Rodentia: Cricetidae), the enzootic reservoir [6]. The virus is detected in bank voles from northern Scandinavia to the Balkan Peninsula, extending into Russia to the eastern part of its range. Human infection with PUUV may be asymptomatic or progress to a more severe form called *nephropathia epidemica* that is associated with fever and oliguria and may result in acute kidney failure [7]. Epidemics are encountered when bank vole populations increase in response to environmental or ecological conditions (e.g., increased food availability) leading to increased virus prevalence in the rodent community [8,9].

The genetic diversity of PUUV has been well characterised in northern and central European countries, and there exist several distinctive genotypes [6,10]. Few comprehensive studies of PUUV genetic diversity in southern Europe have been performed, with the notable exception of Slovenia [11]. Previous detection of PUUV from bank voles in Austria suggested that they belong to the “Alpe-Adria” (ALAD) lineage that has been described from Croatia, Hungary, and Slovenia [12,13]. We, therefore, investigated human cases of PUUV in Austria over 4 years (2016–2019), noting geographic patterns of incidence. We describe the genetic diversity of viruses in selected patient samples and provide virus sequences taken from rodents that support these phylogeographic clusters.

## 2. Materials and Methods

### 2.1. Patient and Rodent Samples

Reporting of diagnosed human hantavirus infections is mandatory in Austria, and the Center for Virology at the Medical University of Vienna is the national reference laboratory for hantavirus infections. We queried our in-house database to identify cases of PUUV virus infection from 2016 to 2019, including cases that were confirmed by RT-PCR and/or PUUV-reactive antibodies. In some cases, patients or primary care physicians were contacted to provide more detail about travel history. Bank voles were captured as part of sanitation efforts following a large outbreak in southern Austria in 2012. Tissues were dissected and stored individually at −80 °C until processing, as described below.

### 2.2. Virus Sequence Analysis

Samples were selected retrospectively from biobank material that is routinely stored in the Center for Virology, with approval from the institutional ethics committee. Samples were selected in an effort to obtain a wide geographic distribution, prioritizing more recent cases when possible. Total RNA was extracted from patient serum samples using a commercial kit (Nuclisens^®^ easyMag^®^, bioMérieux Austria GmbH, Vienna, Austria), and PUUV S segment RNA was detected and amplified using RT-PCR (OneStep RT-PCR kit, QIAGEN GmbH, Hilden, Germany) with previously published primers and protocols [12] and others designed in-house (Table S1). A nested reaction was performed (HotStarTaq Master Mix kit, QIAGEN GmbH) in nearly every sample to amplify a final product of 616 base pairs (bp), which was then sequenced by the Sanger method. Using similar reagents, a portion of the virus M segment (Gc region) was amplified using previously published primers, A1/C2 in RT-PCR and B1/B2 primers in a nested PCR reaction [7], to obtain and sequence amplicons of approximately 450 bp. For both protocols, we pre-emptively designed and included mixtures of these published primers and the same primers with minor modifications (degenerate bases) based on the availability of more contemporary sequences (Table S1). All resulting PUUV sequences were submitted to GenBank (Accession Numbers MW023666-MW023740, and a full list of sequence metadata is provided in File S1).

Sequences were aligned and compared to previously published PUUV sequences isolated from bank voles found in an online database (GenBank) in MEGA7 [14] (full list of published sequences in File S2 and S3). To compare the sequences obtained in this study to previously published sequences, the sequences were trimmed to match the greatest number of available previously published sequences. The total lengths of 505 bases for the S segment and 405 bases for M correspond to viral genomic positions 397–900 (S segment) and 2165–2570 (M segment). For phylogenetic analyses, the *Tula orthohantavirus* reference strain “Moravia/5302v/95” (Accession Numbers NC_005227 for the S segment and NC_005228 for the M segment) was used as an outgroup. 

Phylogenetic inferences using Bayesian methods were performed in BEAST, identifying the best fitting model using the bModelTest plugin [15,16]. The GTR+G+I substitution model with four gamma categories was used for both the partial S and the partial M segment analyses, which included previously published bank vole-derived PUUV reference sequences. In all cases, the chain length was 10^7^ generations, sampling every 1000 iterations, and the effective sample size for all estimated parameters was >200. The resulting maximum clade credibility (MCC) phylograms were built after a 10% burn-in rate, keeping only trees supported by greater than 65% posterior probability. For all methods, the resulting trees were annotated in TreeGraph2 v. 2.15.0 (beta) [17], and the final trees were prepared in MEGA7.

### 2.3. Phylogeographic Analysis

Using patient-reported postal code or specific rodent collection sites, location data were converted to decimal degree latitude and longitude, accurate to one-tenth of a decimal degree (within approximately 11 km), and are thus not explicitly identifiable to maintain patient confidentiality. Geographic visualization was performed in QGIS (v3.16) or in R [18] using packages described below.

Phylogenetic distances were estimated by calculating cophenetic distances (a.k.a. patristic distances or tree distances) using the R package ape (v. 5.0 [19]) based on the MCC tree topology for the partial S segment using all sequences (File S2). Pairwise geographic (geodesic) distances were calculated from decimal degree locations of the sequenced Austrian patient samples and five bank voles from Austria, 2012, in the R package “geosphere” (v. 1.5–10 [20]). Phylogeographic relationships were tested by estimating the correlation between genetic distance and geographic distance using Mantel’s Z, based on a likelihood function comparing pairwise matrices over 10^5^ permutations. This method has previously been used to test the geographic-genetic correlation for zoonotic RNA viruses [21,22,23] including PUUV [24,25,26].

## 3. Results

### 3.1. Geographic Distributrion of Puumala orthohantavirus Cases in Austria

**Figure 1:**
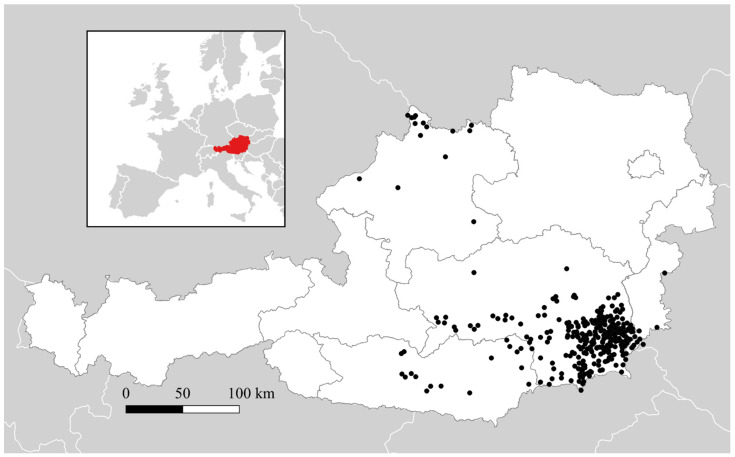
Map of *Puumala orthohantavirus* infections in human patients in Austria, 2009–2019. The inset map shows Austria (red) within Europe. Black dots show the location of 506 georeferenced, confirmed patient infections out of approximately 900 officially reported cases. Austrian Federal States are outlined in the larger map, with a large concentration of cases in South-eastern Styria and a smaller cluster in northern Upper Austria.

On average, from 2009–2019, approximately 60 cases per year were registered in Austria, with notably increased case counts in 2012 (*n* = 264), 2014 (72), 2017 (87), and 2019 (272). Most cases were reported in the south-eastern federal state of Styria, although a second geographically separate group of cases was observed in the district of Rohrbach, in Upper Austria, near the border with Germany and the Czech Republic (Figure 1).

### 3.2. PUUV Genetic Characterization

**Figure 2:**
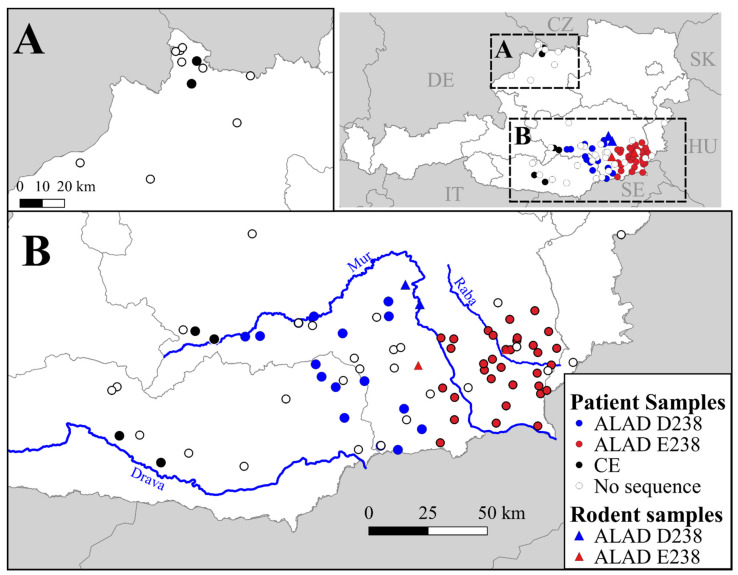
Maps of Austria showing approximate locations of *Puumala orthohantavirus* (PUUV) sequences. In the upper right panel, Austria is shown in white with grey borders around Federal States, and surrounding countries are coloured grey with white country borders labelled with two-letter country codes (CZ = Czech Republic, DE = Germany, HU = Hungary, IT = Italy, SE = Slovenia, SK = Slovakia). The two regions outlined in dashed squares are shown on a larger scale in panels **A**: a region in north central Austria, and **B**: a large cluster in South-eastern Austria. Human patient samples are shown as coloured dots according to genotype: two Alpe-Adria (ALAD) genotypes are shown in red or blue with respect to a nonsynonymous substitution in the nucleocapsid protein at site 238 (D238, blue; E238, red); locations of Central European (CE) genotypes are shown in black. Sample locations from which no sequence was obtained are shown as white dots. Origins of five sequenced rodent samples (*Clethrionomys glareolus*) are shown as triangles coloured as described above, with the easternmost pair overlapping.

We focused our analysis on relatively recent patient samples in our biobank. Of the 412 officially reported cases in 2016–2019, 365 samples were georeferenced and available as biobank material. From these, we selected 93 serum samples for the detection and sequencing of PUUV nucleic acid by a nested RT-PCR (small coloured dots, Figure 2). The samples were selected to provide relatively wide, non-overlapping geographic coverage of southern Austria (Figure 2B), and not strictly based on incidence. We also included four samples from the small cluster of cases in the district of Rohrbach in the northern Federal State of Upper Austria (Figure 2A). We successfully obtained and sequenced amplicons from 57 total samples, including two samples from the Rohrbach district. We also sequenced amplicons from five bank voles captured in 2012 as part of sanitation efforts following the large outbreak during that year (Figure 2B).

**Figure 3:**
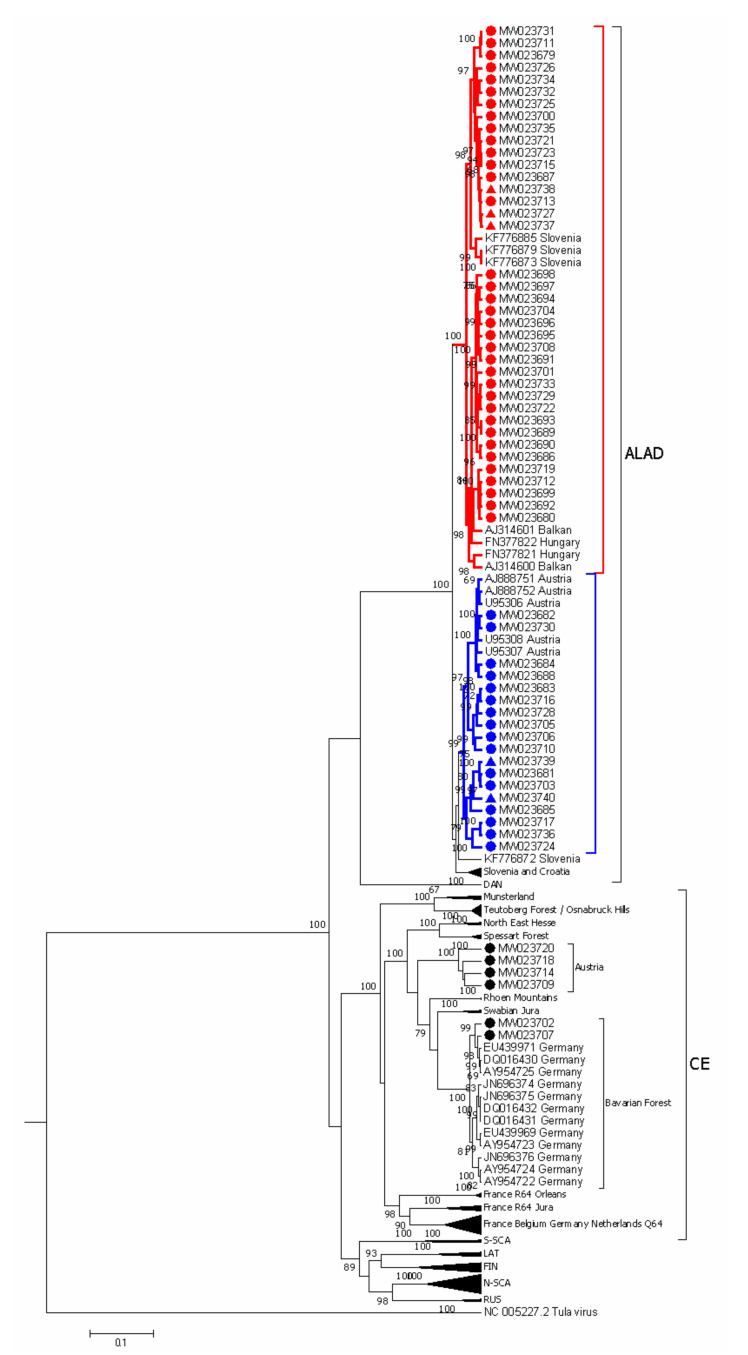
Phylogenetic tree (maximum clade credibility) of partial *Puumala orthohantavirus* (PUUV) S segment sequences (505 nt) from across Europe. Sequences derived from human patients (filled circles) or *Clethrionomys glareolus* (filled triangles) in Austria (labelled with GenBank Accession numbers) are compared to previously published sequences from *C. glareolus* (labelled with GenBank Accession numbers and country of origin). Major established lineages are shown for collapsed branches (e.g., “Danish” = DAN, “Russian” = RUS, “North-Scandanavian” = N-SCA, “South-Scandanavian” = S-SCA, “Finnish” = FIN, “Latvian” = LAT). Sequences from this study are shown within the partially expanded Alpe-Adria (ALAD) and Central Europe (CE) lineages, labelling previously established sublineages of CE lineage (e.g., Swabian Jura, Rhön Mountains, Spessart Forest, etc) according to [27]. We identified two genotypes of ALAD sequences within Austria as defined by cophenetic distances <0.07 and a conserved deduced amino acid substitution in the nucleocapsid protein (E238, red; D238, blue). We identified two sublineages of CE sequences in human patients in Austria: the previously established Bavarian Forest and the herein newly described “Austria” sublineage. *Tula orthohantavirus* reference sequence (NC_005227.1) was used as an outgroup, and the phylogeny was inferred based on the GTR+G+I substitution model with four gamma categories over 10^7^ iterations, sampling every 1000 iterations with a burn-in rate of 10%. Numbers beside branches indicate posterior probability for all nodes with ≥65% support.

First, samples were compared to PUUV sequences isolated from bank voles available in GenBank by constructing a phylogram (Figure 3). With the exception of six samples, all sequences were most similar to the ALAD lineage of PUUV, with high pairwise identity to sequences from Austria, Slovenia, Croatia, and Hungary (>98% identity). Among the six sequences, not matching the ALAD lineages, two samples from the Rohrbach district of Upper Austria were most closely related to the previously characterized “Bavarian Forest” lineage within the Central European (CE) genotype of PUUV [27,28] (Figure 3). Additionally, four samples were also within the CE genotype of PUUV but were not closely related to any previously characterized lineage (“Austria”, Figure 3).

Sequences identified as ALAD lineage had 94% pairwise nucleotide identity on average yet had only 83% mean pairwise identity to the CE lineage sequences. All had the “signature” ALAD deduced amino acid substitutions at V236 and P257 identified by [8], and additionally S233 and I251 were conserved within these sequences.

**Figure 4:**
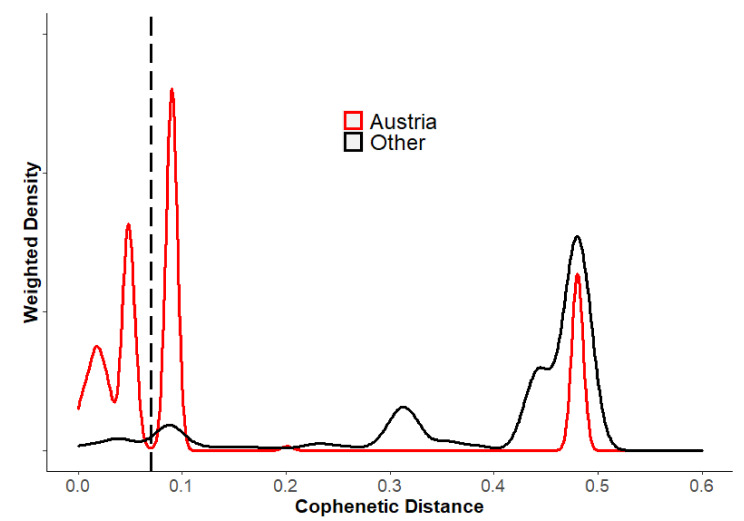
Probability density curves of cophenetic distances based on partial *Puumala orthohantavirus* (PUUV) S segment maximum clade credibility tree. The phylogenetic tree was based on 144 partial (505 nt) sequences of PUUV S segments from rodent reservoirs (*Clethrionomys glareolus*) across Europe, including five from *C. glareolus* and 57 from human patients in Austria, 2012–2019, using the GTR+G+I substitution model. The curves are smoothed histograms showing the pairwise cophenetic distance densities from the subset of Austrian sequences (red) and all other sequences (black, File S2 excluding the reference sequence (*Tula orthohantavirus*, NC_005228, distance >1.0). The curve height is scaled (“weighted density”) for ease of visualisation and comparison. The dashed vertical line shows the threshold of 0.07 used to differentiate the two genotypes of Alpe-Adria PUUV described in Austria.

It was previously shown that cophenetic distances were informative in classifying the genetic diversity of PUUV and related hantaviruses [25,27]. Using this measure of genetic distance, our data further supported the existence of two major lineages in Austria (ALAD and CE); however, we found further genetic evidence of individual genotypes within Austria for both ALAD and CE lineages (Figure 4). Inspecting the distribution of distances for the subset of PUUV that excludes Austrian sequences described herein (“Other”, Figure 4), at least three discrete clusters of distances are discernible: from 0.4–0.6, from 0.2–0.4 (with a peak around 0.31), and <0.20 (Figure 4). For the distribution of distances of sequences described here (“Austria, Figure 4) there are similar discrete clusters (i.e., at ~0.48 and 0.20 distance units); notably there are at least three discrete distance clusters <0.10, the first with a mean of 0.09, the second with a mean of 0.035. These two discrete groups of sequences were separated by a local minimum of approximately 0.07 distance units.

**Figure 5:**
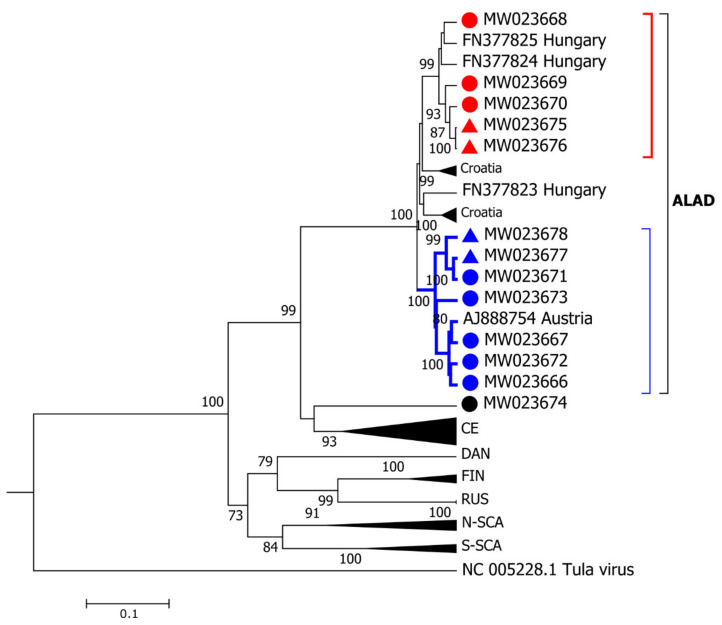
Phylogenetic tree (maximum clade credibility) of partial *Puumala orthohantavirus* (PUUV) M segments. The phylogenetic tree was inferred from 405 nt amplicons of PUUV M segments from human-patient material (*n* = 9, filled circles) or reservoir host lungs (*Clethrionomys glareolus*, filled triangles) using the GTR+G+I substitution model with four gamma categories. The closely related *Tula orthohantavirus* reference strain (NC_005228.1) was used as an outgroup. Posterior probabilities (as percent) supporting each node are displayed for all nodes with >65% support. Previously established lineages are collapsed and labelled (e.g., DAN, FIN, RUS, N-SCA, S-SCA). PUUV sequences from this study (labelled with GenBank Accession number) were identified as either Alpe-Adria (ALAD, bracket) or Central Europe lineage (CE), based on identity of previously published sequences (labelled with Accession number and country, or collapsed and labelled by country). Two ALAD genotypes were identified from the S segment phylogeny, pairwise evolutionary distances, and a deduced amino acid substitution at site 238 in the nucleocapsid protein; shown here in red (E238) and blue (D238). A potentially novel genotype of CE was discovered in the Austrian federal state of Carinthia (accession number MW023674).

The two genotypes of ALAD in Austria separated below a cophenetic distance cut-off of 0.07 (dashed line, Figure 4) had mean within-group pairwise identity of 97% and 96%, respectively, and mean between group similarity of 90%. These two genetic clusters had high node support in both the S segment (Figure 3) and M segment (Figure 5) with over 99% of trees having this separation. Additionally, there were conserved differences in the deduced amino acid of the nucleocapsid protein (N) at position 238, present in both the human- and rodent-derived sequences: sequences from one ALAD sublineage had an aspartic acid (D238) and the other the glutamic acid (E238) that is present in most PUUV sequences. This difference was also noted in previously published ALAD samples: sequences from Slovenia and Croatia have N: D238, while those from Hungary have N: E238. All substitutions in the partial M segment were synonymous. We were, therefore, interested in whether there was geographic support for these genetic clusters.

With only six sequences from the CE lineage, defining genetic clusters is less clear. However, the two samples from Rohrbach, Upper Austria, were clearly within the “Bavarian Forest” group, as defined by others [4,29,30] (Figure 3). The remaining four CE partial S sequences did not fit well within a phylogenetic analysis of previously published CE sequences (Figure 3) with an average of 94% sequence identity to each other, and only 73% sequence identity to the other two CE lineage “Bavarian forest” sequences (Figure 4). Cophenetic distances between these two CE genotypes, based on the partial S segment, were 0.20 units, on average. The phylogenetic analysis of the single partial M sequence obtained from this group also suggests that it likely shares a common ancestor with other CE lineages (Figure 5). Again, there were no differences in the deduced amino acid sequence of the partial M segment, which could be used to differentiate ALAD from CE, nor provide a “signature” for various genotypes within either lineage. Given the convention of naming CE sublineages by locale, we refer to this CE sublineage as “Austria” here; however, there are insufficient data to support a robust phylogeographic analysis of the CE sublineages in Austria at this time (relatively short partial sequences were analysed, no sequences were available from the enzootic host, and comparatively few reference sequences were available for comparison).

### 3.3. Phylogeographic Analysis

As the genetic analysis suggested, the existence of two sublineages each of ALAD and CE in Austria, we tested whether the phylogenetic (cophenetic) and geographic (geodesic) distances of the sequenced S segments (i.e., only those from Austria included in this study) were correlated. The Mantel test, comparing pairwise distance matrices, indicated that phylogenetic distances (Figure 4) and geographic distances (Figure 2) were correlated (Z = 4846.98, *p* < 0.001). Thus, we found strong evidence that the sequences are highly spatially correlated (Figure S1).

Of the 51 samples identified as ALAD lineage, by S segment sequences, 35 were located to the southeast of Graz with the Mur river valley, forming the approximate western border, and the Rába river valley, forming the approximate eastern border (“E238” genotype, Figure 2B). The remaining 16 were located to the west of the Mur river valley (“D238” genotype, Figure 2B). The four CE-Austria genotype sequences were located in both the Drava river valley near Villach and in the upper Mur river valley near Murau (Figure 2B).

## 4. Discussion

The ALAD lineage of PUUV was previously known to be present in Austria [10,12,13], and we identified two genotypes. All previously published PUUV sequences from bank voles captured in Austria—Austria strains from Klippitztörl and Ernstbrunn (GenBank Accession AJ888751 and AJ888752, respectively) in [13,31], as well as the published sequences in [12]—were all within the phylogenetic cluster containing an N: D238 deduced amino acid substitution. To our knowledge, all known PUUV sequences, with the exception of some ALAD sequences, have a glutamic acid at this position (E238), including strains from the “Balkan” region [10], Hungary [32], and Slovenia (Figure 3). 

Similar genetic analyses of PUUV in neighboring Slovenia also identified multiple genotypes of ALAD sequences, based on partial L segment sequences [11]. Based on the published geographic distribution of the L genotypes, namely two major genotypes near the Austrian border, it is likely that the geographic distribution of our two ALAD genotypes overlap those previously identified by Korva et al. [11]. Thus, the true geographic distribution of both genotypes is much larger, likely extending into Slovenia and Hungary. However, we could not combine the two analyses at this time—geographic data were not available for the S segment sequences from Slovenia that were included in our phylogenetic analysis, and the partial L segment was sequenced for their phylogeographic analysis [11]. 

The third genotype of PUUV-ALAD identified by Korva et al. in southern Slovenia are more closely related to sequences from Croatia [32] (Figure 3) within the Dinaric biogeographic region, and it is, therefore, not surprising that we did not identify this sublineage of ALAD in Austria. Combined, there is more support of at least three genotypes of the PUUV-ALAD lineage based on the partial S segment sequences here and the partial L segment sequences of Korva et al. [11]. Other phylogenetic analyses of PUUV (e.g., focusing on the CE lineage [6,29,30,33]) have also predicted the existence of multiple sublineages of ALAD, but few studies have gone into further detail to focus on PUUV diversity in the Alpe-Adrian region.

To our knowledge, the presence of CE lineage PUUV in Austria has not yet been published; however, the existence of the “Bavarian Forest” sublineage of CE is not surprising, given the geographic proximity [27,34,35] (Figure 1 and Figure 2). Thus the “Bavarian Forest” sublineage seems to be distributed in southeast Germany, southwest Czech Republic, and here we report the presence from two sequenced isolates from patients in the Upper Austrian district of Rohrbach (Figure 2A). Seasonal outbreaks are known to occur from this area in Austria, although at a much lower level of incidence than in southern Austria. As the existence of CE-Bavarian Forest in this region was expected, we did not pursue the sequencing of any further isolates from this region.

We identified a potentially novel genotype of PUUV within the CE lineage in Austria, distinct from the “Bavarian forest” sublineage, both phylogenetically and geographically, in a region of typically low PUUV incidence. Notably, these four patient-derived sequences were geographically separated from other reported CE lineages by the Alps—potentially a significant ecological barrier for the enzootic host. The phylogenetic positions, based on the four partial S segment sequences and one partial M segment sequence, were not well resolved (Figure 3 and Figure 5). However, the sequences were decidedly separate from the ALAD sequence cluster, with higher sequence similarity to other CE strains. We believe this provides an interesting group to include, on larger analyses of PUUV phylogeography, with the consideration of the distribution and post-glacial expansion of the reservoir host [6,36]. 

We acknowledge that the M segment sequences, in general, did not provide the best support for the phylogeographic analysis here. Given the proximity of the CE-Austria sublineage isolates to the ALAD isolates, and distinctive geographic separation from other CE sublineage isolates, we were primarily interested in detecting whether reassortment had taken place between the S or M segments of these lineages. We did not detect reassortment. Our analysis of the CE-lineage strains was limited by the facts that (i) we obtained a single M segment sequence from this group of four CE-lineage samples, (ii) we did not have rodent samples from this region, and (iii) we used a relatively short partial sequence of the M segment. The region of the M segment was selected for sequencing based on the availability and efficacy of published nested primer pairs, and not with respect to an abundance of previously published sequences. Nonetheless, we proceeded to analyze this comparatively small genetic sequence of the M segment—note that this carries a large caveat for our interpretation of the data and limits the power of our phylogenetic inferences [37]. Future efforts will focus on obtaining longer sequences from these samples, and also on obtaining virus isolates from the reservoirs in this region.

There have been several methods published to statistically approach phylogeographic analyses (i.e., establishing genetic–geographic correlations with or without temporal resolution). For PUUV, it has been established that genetic diversity can largely be attributed to the isolation-by-distance pattern of divergence, owing to the relatively small dispersal ranges of the enzootic host [9,25,26,38]. Our data also support the strong correlation between genetic and geographic distance on a small scale for PUUV, using cophenetic distance as the measure of phylogenetic distance. In general, our phylogenetic analysis of the partial S segment suggested that there exist discrete distances between groups of PUUV (Figure 4), and closer inspection shows that these groups are largely based on geography. For example, distances greater than 0.4 (for this substitution model, with these selected sequences, over this partial S segment sequence) separated PUUV into three clades: southern (ALAD), central (CE), and northern (RUS, N-SCA, S-SCA, FIN, LAT), with respect to their distribution in Europe (Figure 3). Notably, the strain “Fyn” (the so-called “DAN” lineage from Denmark) did not follow this pattern, and grouped with the more southern ALAD viruses, which may be an artefact of Bayesian vs. Maximum likelihood methods of phylogenetic inference, as noted by [6]. Another group of distances was observed between 0.2 and 0.4 distance units (Figure 4), and separated lineages within the “northern” clades and “sub-lineage groups” within the CE clade (Figure 3). A threshold of 0.21 was chosen by Faber et al. [27] to delineate sublineages of CE within Germany using a nearly identical genetic region as used in our analysis, and this supported the previously used naming conventions for CE sublineages. However, extending the cut-off to 0.20 would group the southern German sublineages (Bavarian Forest and Swabian Jura) into one sublineage that would also include both CE genotypes described here (the distance between Bavarian Forest and the “new” Austrian genotype” ~0.201). Again, these analyses are based on incomplete genetic information, and require more detailed formal analyses. 

Our phylogenetic analysis of the partial S segment sequences showed that all known (published) ALAD sequences have an average distance of 0.065, and a maximum distance of 0.09 (for the partial S segment using stated substitution rate, Figure 4). Thus, ALAD S segments appear to be less diverse than CE as a whole. However, we note that comparing the geographic area covered by published CE strains is much larger than that covered by published ALAD sequences (e.g., map in [6]). This may account for the lower genetic diversity under the isolation-by-distance assumption (c.f. Figure S1 to similar curves in [25,26]). Even so, others have identified potentially three distinctive phylogeographic groups of PUUV-ALAD (by partial L segment [11]), and we show that at least two genotypes exist in Austria—identifiable by a conserved predicted amino acid substitution in the nucleocapsid protein, with clear separation at a cophenetic distance of <0.07 (i.e., below the maximum diversity value for ALAD of ~0.1). In fact, inspecting the smoothed histogram of Austria-only sequences, one can see the distances between ALAD and CE (~0.32), the distances between CE genotypes (a small peak at ~0.2), and three peaks <0.1 distance units that correspond to within-Austria ALAD distances (Figure 4). 

Having defined these genetic clusters, we attempted several methods to test whether cases, themselves, were geographically clustered. We made the assumption that patients reported relatively accurate probable infection locations, but obviously such genetic–geographic analyses would be ideally performed with the enzootic host. Several unsupervised clustering algorithms (e.g., k-means clusters, hierarchical clusters, and NMDS ordination) were tried but did not provide the accurate assessment of genotype (based on confusion matrix analysis). Additionally, we attempted supervised clustering using the genotype locations to construct 95% data ellipses around their centroids. This resulted in overlapping regions, ambiguously classifying approximately 8% of the human cases known from the region. Thus, the locations did not seem to form discrete geographic clusters, although there was clearly genetic–geographic correlation and the genotypes are sorted geographically (Figure 2B).

For sequences less than 0.07 distance units apart, there is another cluster of sequences with mean pairwise cophenetic distances at 0.035 (Figure 4). This represents further subdivisions of the two major ALAD genotypes (at approximately the same distances within genotypes, 0.032 and 0.038, respectively), into two or three minor genotypes. These minor genotypes also appeared to be geographically clustered. However, our analysis only included sequences derived from bank voles within the two genotypes we describe in detail (threshold genetic distance of 0.07, with observed nonsynonymous substitution in the nucleocapsid protein), and we, therefore, did not classify these finer scale phylogeographic clusters here. In addition to using partial genetic sequences, the source of our viral sequences (i.e., patient samples and few rodent hosts samples) brings another large caveat to our study design, and more specifically a lack of power by using patient-reported postal codes to assign location. While this prevents us from performing robust geographic analyses, we note that the genetic analysis could detect genetic diversity at a rather fine level of detail.

## Data Availability

The data presented in this study are available from the corresponding author upon reasonable request.

## Supplementary Materials

The following are available online at https://www.mdpi.com/article/10.3390/v13040640/s1, Table S1. List of primers used for amplification of *Puumala orthohantavirus* sequences from human patients and rodents in Austria. Positions are in relation to the NCBI Reference sequence (GenBank Accession numbers for S segment, NC_005224.1, and M segment, NC_005223.1). Modifications to published primers are given asterisks. Figure S1. Pairwise genetic distance between partial *Puumala orthohantavirus* (PUUV) S segment sequences obtained from Austrian patients and bank voles plotted against pairwise geodesic distance. Genetic distances are measured by calculating the cophenetic distance based on a Bayesian maximum clade credibility phylogram (based on a GTR+G+I substitution model) of a large selection of PUUV sequences available in GenBank (File S2). This figure shows a subset of only the georeferenced samples included in our study (File S1), from Austria 2012–2019. File S1. List of *Puumala orthohantavirus* sequences retrieved from patients and rodents in Austria deposited in GenBank. Accession number is followed by location (“Austria”_Federal State), isolate number (“Hu” = human serum-origin, “Mg” = *Clethrionomys glareolus* lung), and year of collection separated by forward slashes. The viral genomic segment is indicated at the end. File S2. List of reference *Puumala orthohantavirus* S segment sequences used in phylogenetic analysis. All sequences were reported from the reservoir host, *Clethrionomys glareolus*, accessed from GenBank sequence database. Accession numbers are followed by Country and locale, isolate, and year (when available) separated by forward slashes. File S3. List of reference *Puumala orthohantavirus* M segment sequences used in phylogenetic analysis. All sequences were reported from the reservoir host, *Clethrionomys glareolus*, accessed from GenBank sequence database. Accession numbers are followed by country, isolate, and year (when available) separated by forward slashes.
