# Supplementary material for: Genetic Diversity of Puumala orthohantavirus in Rodents and Human Patients in Austria, 2012–2019
Source: Viruses. 2021 Apr 8;13(4):640. doi: 10.3390/v13040640 (PMC8069475; doi:10.3390/v13040640)
Supplement: Supplementary file 1 [file viruses-13-00640-s001.zip › viruses-1152312-supplementary.pdf]

Supplemental Table 1. List of primers used for amplification of *Puumala orthohantavirus* sequences from human patients and rodents in Austria. Positions are in relation to the NCBI Reference sequence (GenBank Accession numbers for S segment, NC_005224.1, and M segment, NC_005223.1). Modifications to published primers are given asterisks.

Primer	Sequence	Segment	Positions	Citation
PuuALAD_F	ACCAYCTCAAGGAGCGATCA	S	305-324	In house
PuuALAD_R	ACCWAGTTCAGCCATCCCTG	S	1022-1041	In house
PuuRBBF_F	ACCAYCTYAAGGARCGATCA	S	305-324	In house
PuuRBBF_F	ACCWAACTCTGCCATTCCTG	S	1022-1041	In house
PPT334C	TATGGIAATGTCCTTGATGT	S	334-353	Bowen et al. 1997
PPT334C*	TATGGAAATGTCCTTGATGT	S	334-353	In house
PPT986R	GCACATGCAAATACCCA	S	970-986	Bowen et al. 1997
PPT986R*	GCACATGCRAATACCCA	S	970-986	In house
PUUV_A1	AATCCATCTGAGGCTACACCGTCT	M	1793-1816	Plyusnin et al. 1997
PUUV_A1*	AATCCTGCTGAAGCHACAACATC	M	1793-1815	In house
PUUV_C2	CCAACTCCTGAACCCCATGC	M	3003-3021	Plyusnin et al. 1997
PUUV_B1	AACCCGGCAAATGAACAAGAA	M	2147-2167	Plyusnin et al. 1997
PUUV_B2*	TTGTTCAAGTGGACCTARAAAT	M	2611-2632	In house

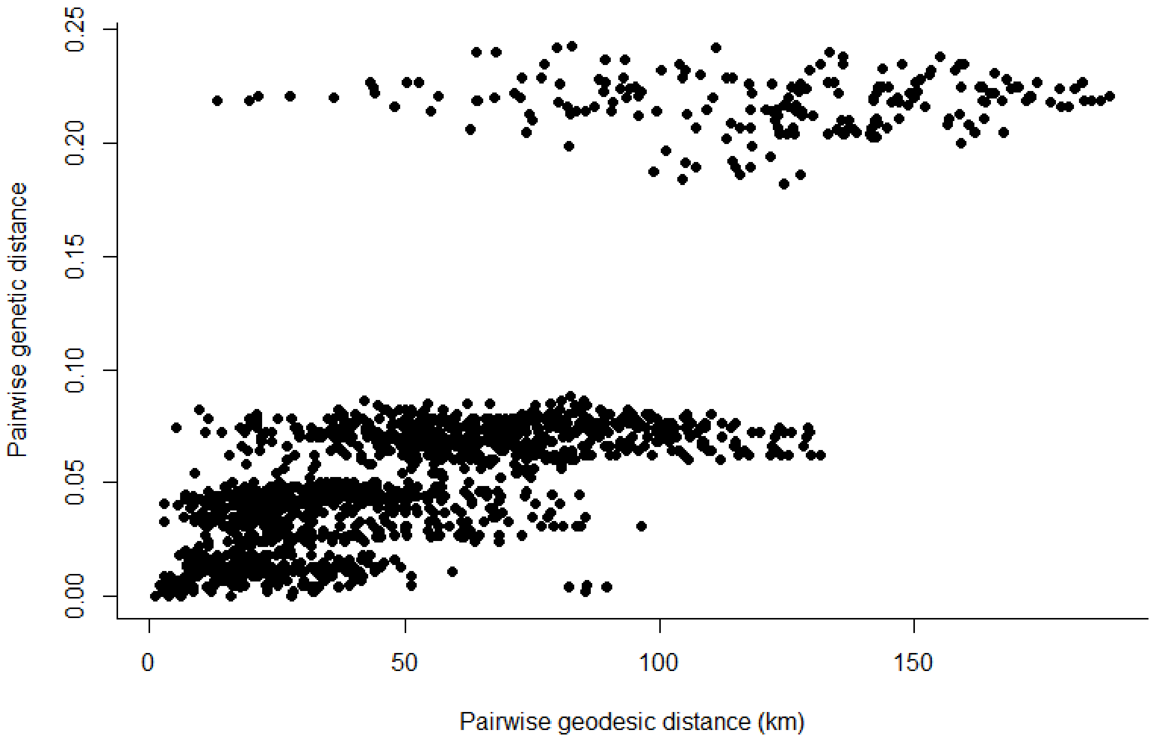

Supplemental Figure S1. Pairwise genetic distance between partial *Puumala orthohantavirus* (PUUV) S segment sequences obtained from Austrian patients and bank voles plotted against pairwise geodesic distance. Genetic distances are measured by calculating the cophenetic distance based on a Bayesian maximum clade credibility phylogram (based on a GTR+G+I substitution model) of a large selection of PUUV sequences available in GenBank (Supplemental Data 2). This figure shows a subset of only the georeferenced samples included in our study (Supplemental Data 1), from Austria 2012-2019.

Supplemental Data 1. List of *Puumala orthohantavirus* sequences retrieved from patients and rodents in Austria deposited in GenBank. Accession number is followed by location ("Austria"_Federal State), isolate number ("Hu" = human serum-origin, "Mg" = *Clethrionomys glareolus* lung), and year of collection separated by forward slashes. The viral genomic segment is indicated at the end.

MW023666 Austria_Carinthia/Hu1095139/2018 M segment
MW023667 Austria_Carinthia/Hu1105858/2018 M segment
MW023668 Austria_Styria/Hu1141379/2019 M segment
MW023669 Austria_Styria/Hu1145193/2019 M segment
MW023670 Austria_Styria/Hu1153799/2019 M segment
MW023671 Austria_Styria/Hu1155512/2019 M segment
MW023672 Austria_Styria/Hu1161395/2019 M segment
MW023673 Austria_Styria/Hu1197818/2019 M segment
MW023674 Austria_Carinthia/Hu1197858/2019 M segment
MW023675 Austria_Styria/Mg14_Mitterfladnitz/2012 M segment
MW023676 Austria_Styria/Mg16_ Mitterfladnitz/2012 M segment
MW023677 Austria_Styria/Mg18_Gamsgraben/2012 M segment
MW023678 Austria_Styria/Mg9_Peggau/2012 M segment
MW023679 Austria_Styria/Hu1025550/2017 S segment
MW023680 Austria_Styria/Hu1026374/2017 S segment
MW023681 Austria_Styria/Hu1028716/2017 S segment
MW023682 Austria_Carinthia/Hu1029394/2017 S segment
MW023683 Austria_Carinthia/Hu1095139/2018 S segment
MW023684 Austria_Carinthia/Hu1105858/2018 S segment
MW023685 Austria_Styria/Hu1116851/2019 S segment
MW023686 Austria_Styria/Hu1120604/2019 S segment
MW023687 Austria_Styria/Hu1124640/2019 S segment
MW023688 Austria_Styria/Hu1128698/2019 S segment
MW023689 Austria_Styria/Hu1130869/2019 S segment
MW023690 Austria_Styria/Hu1135128/2019 S segment
MW023691 Austria_Styria/Hu1136110/2019 S segment
MW023692 Austria_Styria/Hu1137421/2019 S segment
MW023693 Austria_Styria/Hu1138226/2019 S segment
MW023694 Austria_Styria/Hu1139352/2019 S segment
MW023695 Austria_Styria/Hu1140233/2019 S segment
MW023696 Austria_Styria/Hu1141379/2019 S segment
MW023697 Austria_Styria/Hu1141709/2019 S segment
MW023698 Austria_Styria/Hu1143892/2019 S segment
MW023699 Austria_Styria/Hu1145193/2019 S segment
MW023700 Austria_Styria/Hu1147347/2019 S segment
MW023701 Austria_Styria/Hu1147348/2019 S segment
MW023702 Austria_Upper Austria/Hu1154432/2019 S segment
MW023703 Austria_Styria/Hu1155512/2019 S segment
MW023704 Austria_Styria/Hu1161372/2019 S segment
MW023705 Austria_Styria/Hu1161395/2019 S segment
MW023706 Austria_Styria/Hu1166857/2019 S segment
MW023707 Austria_Upper Austria/Hu1171197/2019 S segment
MW023708 Austria_Styria/Hu1176890/2019 S segment
MW023709 Austria_Styria/Hu1177866/2019 S segment
MW023710 Austria_Styria/Hu1177869/2019 S segment
MW023711 Austria_Styria/Hu1178867/2019 S segment
MW023712 Austria_Styria/Hu1180564/2019 S segment

MW023713 Austria_Styria/Hu1182969/2019 S segment
MW023714 Austria_Styria/Hu1185874/2019 S segment
MW023715 Austria_Styria/Hu1191554/2019 S segment
MW023716 Austria_Styria/Hu1193871/2019 S segment
MW023717 Austria_Styria/Hu1197818/2019 S segment
MW023718 Austria_Carinthia/Hu1197858/2019 S segment
MW023719 Austria_Styria/Hu1198609/2019 S segment
MW023720 Austria_Carinthia/Hu1206487/2019 S segment
MW023721 Austria_Styria/Hu1209813/2019 S segment
MW023722 Austria_Styria/Hu920792/2016 S segment
MW023723 Austria_Styria/Hu938383/2016 S segment
MW023724 Austria_Styria/Hu946014/2016 S segment
MW023725 Austria_Styria/Hu986729/2017 S segment
MW023726 Austria_Styria/Hu995423/2017 S segment
MW023727 Austria_Styria/HuML33_Mi/2012 S segment
MW023728 Austria_Styria/Hu1146291/2019 S segment
MW023729 Austria_Styria/Hu1124641/2019 S segment
MW023730 Austria_Carinthia/Hu1154531/2019 S segment
MW023731 Austria_Styria/Hu1153799/2019 S segment
MW023732 Austria_Styria/Hu1176203/2019 S segment
MW023733 Austria_Styria/Hu1176206/2019 S segment
MW023734 Austria_Styria/Hu1176856/2019 S segment
MW023735 Austria_Styria/Hu1177196/2019 S segment
MW023736 Austria_Styria/Hu1195950/2019 S segment
MW023737 Austria_Styria/Mg14_Mitterfladnitz/2012 S segment
MW023738 Austria_Styria/Mg16_ Mitterfladnitz /2012 S segment
MW023739 Austria_Styria/Mg18_Gamsgraben/2012 S segment
MW023740 Austria_Styria/Mg9_Peggau/2012 S segment

Supplemental Data 2. List of reference *Puumala orthohantavirus* S segment sequences used in phylogenetic analysis. All sequences were reported from the reservoir host, *Clethrionomys glareolus*, accessed from GenBank sequence database. Accession numbers are followed by Country and locale, isolate, and year (when available) separated by forward slashes.

AB297665 Russia Bashkiria/DTkUfa-97/1997
AF367066 Russia Omsk/CG215/2000
AJ223368 Norway Eidsvoll/1124v/1987
AJ223369 Norway Eidsvoll/Cg1138/1987
AJ223371 Norway Huggberget/Cg36/1994
AJ223374 Norway Mellansel/Cg47/1994
AJ223375 Norway Mellansel/Cg49/1994
AJ223377 Norway Solleftea/6Cg/1994
AJ223380 Norway Tavelsjo/Cg81/1994
AJ238779 Germany Cg-Erft//1999
AJ238788 Russia Karhumaki//1995
AJ238789 Russia Kolodozero//1995
AJ238790 Russia Gomselga//1995
AJ238791 Denmark Fyn//1990
AJ277030 Belgium Thuin/33Cg/1996
AJ277032 Belgium Momignies/47Cg/1996
AJ277034 Belgium Couvin/59Cg/1996
AJ277076 Belgium Montbliart/CG14445/

AJ314597 Finland Pallasjarvi/63Cg/1998
AJ314600 Balkan /65Cg/2000
AJ314601 Balkan /78Cg/2000
AJ888751 Austria Klippitztoerl/Cg9/1995
AJ888752 Austria Ernstbrunn/Cg641/1995
AM695638 France Mignovillard/CgY02/2005
AM746299 Sweden Bussjo/95-3/1995
AM746310 Sweden Djaknebole/98-1998/1998
AM746315 Sweden Gumboda/98-1/1998
AM746316 Sweden Gumboda/98-2/1998
AM746320 Sweden Norum/98-1/1998
AM746324 Sweden Norum/98-5/1998
AM746325 Sweden Norum/98-6/1998
AM746330 Sweden Palbole/98-4/1998
AY954722 Germany Bavaria/CG_9/2004
AY954723 Germany Bavaria/CG_34/2004
AY954724 Germany Bavaria/CB_20/2004
AY954725 Germany Bavaria/CG_2/2004
DQ016430 Germany Bavaria/CG_33/2004
DQ016431 Germany Bavaria/CG_39/2004
DQ016432 Germany Bavaria/CG_41/2004
DQ322669 Germany Cologne/Mu05_241/2005
EU266762 Germany Albstadt/M4/2007
EU439969 Germany Bavaria/152/2005
EU439971 Germany Bavaria/Mg157/2005
FN377821 Hungary Mg9_HungaryTR17//2000
FN377822 Hungary Mg23_HungaryTR17//2000
GQ339473 Finland Kiviniemi/Mg3/2005
GQ339476 Sweden Aijajarvi/Mg7/2005
GQ339478 Sweden Jockfall/Mg12/2005
GQ339479 Sweden Moskosel/Mg17/2005
GQ339480 Sweden Gyttjea/Mg19/2005
GQ339481 Sweden Ljustrask/Mg20/2005
GQ339482 Sweden Kalvudden/Mg22/2005
HE801633 Finland Sotkamo//2009
JN657228 Latvia Jelgava/Mg149/2008
JN696357 Germany Sinntal/Mu442/2010
JN696358 Germany Osnabrueck/Mu362/2005
JN696361 Germany Heimerdingen/Mu1083/2010
JN696362 Germany Billerbeck/Mu2034/2010
JN696365 Germany Bramsche/Mu3072/2010
JN696366 Germany Darmstadt/Mu427/2009
JN696367 Germany Darmstadt/Mu428/2009
JN696368 Germany Gilserberg/Mu557/2010
JN696369 Germany Salmuenster/Mu563/2010
JN696370 Germany Weissach/Mu978/2010
JN696373 Germany Karlstadt/MuEb10/2010
JN696374 Germany Lackenberg/MuEb12/2010
JN696375 Germany Lackenberg/MuEb14/2010
JN696376 Germany Elsenthal/MuEb51/2010
JN831943 Finland Pieksamaki/Mg7/2008
JQ319163 Finland Konnevesi/Mg_M94A/2005

JQ319164 Finland Konnevesi/Mg_O6B/2005
JQ319168 Finland Konnevesi/Mg_O22B/2005
KC676609 Croatia Gerovo/Mg938/2008
KC676610 Croatia Gerovo/Mg954/2008
KC676611 Croatia Gerovo/Mg955/2008
KC676612 Croatia Gerovo/Mg978/2008
KC676613 Croatia Gerovo/Mg979/2008
KC676614 Croatia Gerovo/Mg980/2008
KC676615 Croatia Gerovo/Mg982/2008
KF776864 Slovenia /HtSi1054/1995
KF776872 Slovenia /HtSi1101/2007
KF776873 Slovenia /HtSi1069/1999
KF776879 Slovenia /HtSi1063/1999
KF776881 Slovenia /HtSi1058/1995
KF776884 Slovenia /HtSi1079/1995
KF776885 Slovenia /HtSi1071/1999
KJ994776 Germany Astrup/Mu_07_12/2007
KR047244 Germany Ellerbeck/eller_08_025/2008
KR047245 Germany Ellerbeck/eller_10_028/2010
KR047257 Germany Schledehausen/schle_08_053/2008
KR047259 Germany Schledehausen/schle_08_059/2008
KR047313 Germany Bramsche/varus_12_032/2012
KT247593 France Ardennes/Mg75/2011
KT247594 France Orleans/Mg23/2010
KT247595 France Orleans/Mg29/2010
KT247596 France Jura/Mg2/2010
KU314900 Netherlands A/Mg1012/2010
KU314901 Netherlands A/Mg1439/2011
KU314902 Netherlands B/Mg2/2008
KU314905 Netherlands E/Mg591/2008
KU670632 Germany Goettingen/KS11_1342/
KU670634 Germany Cologne/KS11_2387/
KU670635 Germany Osnabrueck/KS14_715/
KU670636 Germany Osnabrueck/KS14_833/
KU670637 Germany Osnabrueck/KS14_873/
KU670638 Germany Osnabrueck/KS14_766/
KU670639 Germany Osnabrueck/KS14_778/
KU670640 Germany Osnabrueck/KS10_3078/
KU670641 Germany Walbeck/Mu07_116/
KU670642 Germany Diedorf/KS10_3482/
KU670643 Germany Diedorf/KS10_3485/
KX757839 Lithuania /LT15_164/2015
KX757840 Lithuania /LT15_174/2015
KX757841 Lithuania /LT15_201/2015
KX815394 Poland /KS13_855/2009
KY365000 France Murbach-Alsace/2012_2/2012
MG812389 Belgium Gierle/MG3/2012
MG812391 Belgium Chimay/MG1/2012
MG812393 Belgium Rance/MG1/2012
MG812395 Belgium Beaumont/MG1/20
MG812434 Belgium Beaumont/MG9/20
MG812435 Belgium Fontaine-Leveque/MG1/2007

MG812443 Belgium Zevendonk/MG3/2007
MG812457 Belgium Viroinval/MG1/2007
MG812466 Belgium Chimay/MG1/2010
MK946422 France Orleans/NCHA376/2014
MK946423 France Orleans/NCHA380/2014
MK946425 France Septmoncel-Jura/Camp9/2015
MK946427 France Poligny-Jura/NCHA181/2014
MK946428 France Orleans/ORW12_54/2012
MK946429 France Vosges/LPP/2015
MK946430 France Ardennes/CSSP15-5/2015
MK946434 France Ardennes/Hargnies_161/2011
MN026263 Germany Winningen/H104/2018
MN026264 Germany Drensteinfurt/H13025/2018
U14137 “Vranica”
U22423 France CG/13891/1990
U95306 Austria /Kl6/1995
U95307 Austria /P1/1995
U95308 Austria /Kl1/1995
Z21497 Russia Udmurtia/894Cg/1991
Z30704 Finland Evo/14Cg/1993
Z30708 Russia Udmurtia/338Cg/1992
Z48586 Sweden Vindeln/L20Cg/1983

Supplemental Data 3. List of reference *Puumala orthohantavirus* M segment sequences used in phylogenetic analysis. All sequences were reported from the reservoir host, *Clethrionomys glareolus*, accessed from GenBank sequence database. Accession numbers are followed by Country, isolate, and year (when available) separated by forward slashes.

AJ238778 Germany/Cg-Erft/
AJ278091 Denmark Fyn/47/
AJ877240 Croatia Okucani/387/
AJ877241 Croatia Okucani/416/
AJ877242 Croatia Okucani/421/
AJ877243 Croatia Nova Gradiska/730/
AJ877244 Croatia Nova Gradiska/Cg/
AJ888753 Austria Ernstbrunn/Cg64/1995
AJ888754 Austria Klippitztoerl/Cg641/1995
AM695639 France Mignovillard/CgY/2005
FN377823 Hungary HungaryTR17/Mg2/2000
FN377824 Hungary HungaryTR17/Mg9/2000
FN377825 Hungary HungaryTR17/Mg23/2000
GQ339488 Sweden Kiviniemi/Mg3/2005
GQ339489 Sweden Jockfall/Mg12/2005
GQ339490 Sweden Moskosel/Mg17/2005
GQ339491 Sweden Kalvudden/Mg22/2005
GQ339492 Sweden Bergsjobo/Mg25/2005
GQ339493 Sweden Faboviken/Mg26/2005
GQ339494 Sweden Mangelbo/Mg1/2005
GQ339495 Sweden Munga/Mg16/2005
JN831944 Finland Pieksamaki/Mg7/2008
JQ319172 Finland Konnevesi/MgO78A/2005
JQ319247 Finland Konnevesi/MB9/2008-9
KC676629 Croatia Gerovo/Mg938/2008
KC676630 Croatia Gerovo/Mg954/2008
KC676631 Croatia Gerovo/Mg955/2008
KC676632 Croatia Gerovo/Mg978/2008
KC676633 Croatia Gerovo/Mg979/2008
KC676634 Croatia Gerovo/Mg980/2008
KC676635 Croatia Gerovo/Mg982/2008
KJ994777 Germany Osnabrueck/Mu07_1219/20007
KT247598 France Jura/Mg2/2010
KT247599 France Jura/Mg214/2010
KT247600 France Orleans/Mg23/2010
KT247601 France Orleans/Mg29/2010
KT247602 France Ardennes/Mg75/2011
KT247603 France Ardennes/Mg156/2011
KT885051 Russia /CG1820_POR/2015
KU314906 Netherlands /Mg31/2007
KU314907 Netherlands /Mg25/2007
KU314912 Netherlands /Mg2/2008
KU314913 Netherlands /Mg66/2008
L08754 Russia /K27/
Z49214 Sweden Vindeln/L20Cg/1983
Z70201 Finland Virrat/25Cg/1995
